# Does pulmonary subspecialty referral from primary care affect the adherence to vaccination recommendations in COPD patients?

**DOI:** 10.1186/s12931-021-01639-6

**Published:** 2021-02-12

**Authors:** Solmaz Ehteshami-Afshar, Kristina Crothers, Benjamin Rodwin, Brett Bade, Cynthia Brandt, Kathleen M. Akgün

**Affiliations:** 1 Internal Medicine, Yale University School of Medicine, New Haven, CT USA; 2 Veterans Affairs Puget Sound and University of Washington, Seattle, WA USA; 3 Veterans Affairs Connecticut Healthcare System, West Haven, CT USA; 4 Veterans Affairs Connecticut Pain Research, Informatics, Multi-Morbidities, and Education Center, West Haven, CT USA; 5 Emergency Medicine, Yale School of Medicine, New Haven, CT USA

**Keywords:** Chronic obstructive pulmonary disease, Vaccination, Influenza, Pneumococcal vaccine, Referral

## Abstract

The importance of vaccinations for COPD patients has been previously described. However, there is still a gap between guideline recommendations and the implementation of preventive care delivery for these patients. Specially, the rise of SARS-CoV-2 pandemic has made the significance of vaccination adherence more critical to address. Our study showed that referral to pulmonary clinic is associated with increased odds of receiving influenza (OR = 1.97, [95% CI 1.07, 3.65]) and pneumococcal vaccinations (PCV13 OR = 3.55, [1.47, 8.54]; PPSV23 OR = 4.92, [1.51, 16.02]). These data suggest that partnerships between primary care physicians and pulmonologists can potentially improve the vaccination rates for patients with COPD.

## Introduction

Chronic obstructive pulmonary disease (COPD) is one of the most common causes of morbidity and mortality worldwide [1]. Despite the importance of providing guideline-based care, adherence to Global Initiative for Chronic Obstructive Lung Disease (GOLD) recommendations [2] has been sub-optimal, including pharmacologic and non-pharmacologic treatments [3–13]. Preventive treatments with successful vaccinations against lung infections are critical for improving COPD health but adherence can be increased. Vaccination adherence and addressing possible barriers is especially important in the setting of the global respiratory pandemic of SARS-CoV-2. To inform quality improvement targets, we ascertained the adherence to pulmonary infection-related vaccination recommendations in COPD patients, stratified by primary care management alone compared to those with pulmonary subspecialty referral.

## Methods

We performed a retrospective cross-sectional analysis on a random sample of COPD patients receiving primary care (PC) in a single clinical site from January 1, 2018 to December 31, 2019. Patients first diagnosed with COPD during that timeframe were identified by international classification of disease, tenth revision codes (ICD10 = J40–44; n = 3769). We purposefully sampled women (n = 100) and randomly selected 100 men to include in the chart review (5% sample). Our primary outcomes were vaccination for pneumococcal conjugate (PCV13), pneumococcal polysaccharide (PPSV23) and influenza (3 consecutive calendar years 2017–2019), accounting for recommendations in presumably immunocompetent adults with COPD: PCV13 for patients ≥ 65-years-old; PPSV23 for patients ≥ 65-years-old or those with COPD and severely reduced FEV1; influenza for all included COPD patients [2]. We identified vaccination records for PCV13 and PPSV23 (including before the study period), influenza vaccination records (2017–2019), smoking status (current, former, never), age and gender of the patients by detailed chart review of PC and/or pulmonary text notes from the electronic health record.

Descriptive statistics were used to compare demographic and clinical variables by pulmonary subspecialty evaluation: the student's t-test to compare means for continuous variables and Chi-square test for categorical variables. To assess the association between subspecialty pulmonary evaluation with vaccination adherence, multivariable-adjusted logistic regression was performed and odds ratio (OR) with 95% confidence interval (CI) were determined. The model was adjusted for age, gender and smoking status. Statistical significance was defined as p < 0.05. All statistical analyses were done by IBM SPSS statistics version 25.

## Results

Among 200 patients (mean age = 70.5 ± 10.9, 50% women), 78 patients had pulmonary subspecialty evaluation in the prior 3 years. Patients with pulmonary evaluation were more likely to be current smokers (59%), had higher pack-year smoking history (48.4 pack-year vs. 37.7 pack-year respectively, p = 0.01) and were more likely to have had a COPD-related hospitalization in the preceding 12 months (20.5% vs. 2.4%; p < 0.001) (Table 1). Restricting to patients ≥ 65 years old, PCV13 and PPSV23 vaccination rates were significantly higher amongst patients referred to pulmonary (96.6% vs. 81.6%, p = 0.009; 94.8% vs. 84.7%, p = 0.03, respectively). Influenza vaccination rates were higher for patients referred for pulmonary evaluation in all years studied compared to those only managed by PC providers: 2017 = 81% vs. 63%, p = 0.008; 2018 = 80% vs. 62.5%, p = 0.008; and 2019 = 70.5% vs. 52%, p = 0.011, respectively.

**Table 1:** Patients demographics and clinical variables

	No pulmonary clinic visit in prior 3 years (n = 122)	Pulmonary clinic visit in prior 3 years (n = 78)	p value
Age in years (mean ± SD)	70.5 ± 10.8	70.4 ± 10.9	0.9
Age ≥ 65-year-old, n(%)	82 (65.5)	54 (69.2)	0.7
Men, n (%)	56 (45.9)	44 (56.4)	0.2
Smoking status, n (%)			
Never	6 (4.9)	10 (12.8)	0.005
Former	57 (46.7)	20 (25.6)	
Current	56 (45.9)	46 (58.9)	
PFT ever done, n (%)	70 (57.3)	77 (98.7)	< 0.001
Obstructive pattern confirmed on PFT (%)	42 (60.0)	66 (85.7)	< 0.001
FEV1/FVC (mean ± SD)	69.4 ± 11.3	65.1 ± 14.6	0.07
Airflow limitation by FEV1 in those with obstructive pattern, n (%)			
GOLD 1 (mild)	8 (19.0)	7 (10.6)	0.02
GOLD2 (moderate)	28 (66.6)	30 (45.5)	
GOLD 3 (severe)	5 (11.9)	24 (36.4)	
GOLD 4 (very severe)	1 (2.4)	5 (7.5)	
PCV13, n (%)	71/87 (81.6)	57/59 (96.6)	0.009
PPSV23, n (%)	100/118 (84.7)	74/78 (94.8)	0.03
Influenza vaccination 2017, n (%)	77(63.1)	63(80.8)	0.008
Influenza vaccination 2018, n (%)	76(62.8)	62(80.5)	0.008
Influenza vaccination 2019, n (%)	60 (52.2)	53(70.7)	0.01
COPD hospitalization in prior 12 months, n (%)	3 (2.4)	16 (20.5)	< 0.001
Death in 2017–2019, n (%)	8 (6.5)	5 (6.4)	1.0

*COPD* chronic obstructive pulmonary disease, *FEV1* forced expiratory volume in 1 s, *FVC* forced vital capacity, *PFT* Pulmonary Function Testing, *PCV13* pneumococcal conjugate vaccine, *PPSV23* pneumococcal polysaccharide vaccine, *SD* standard deviation

**Fig. 1:**
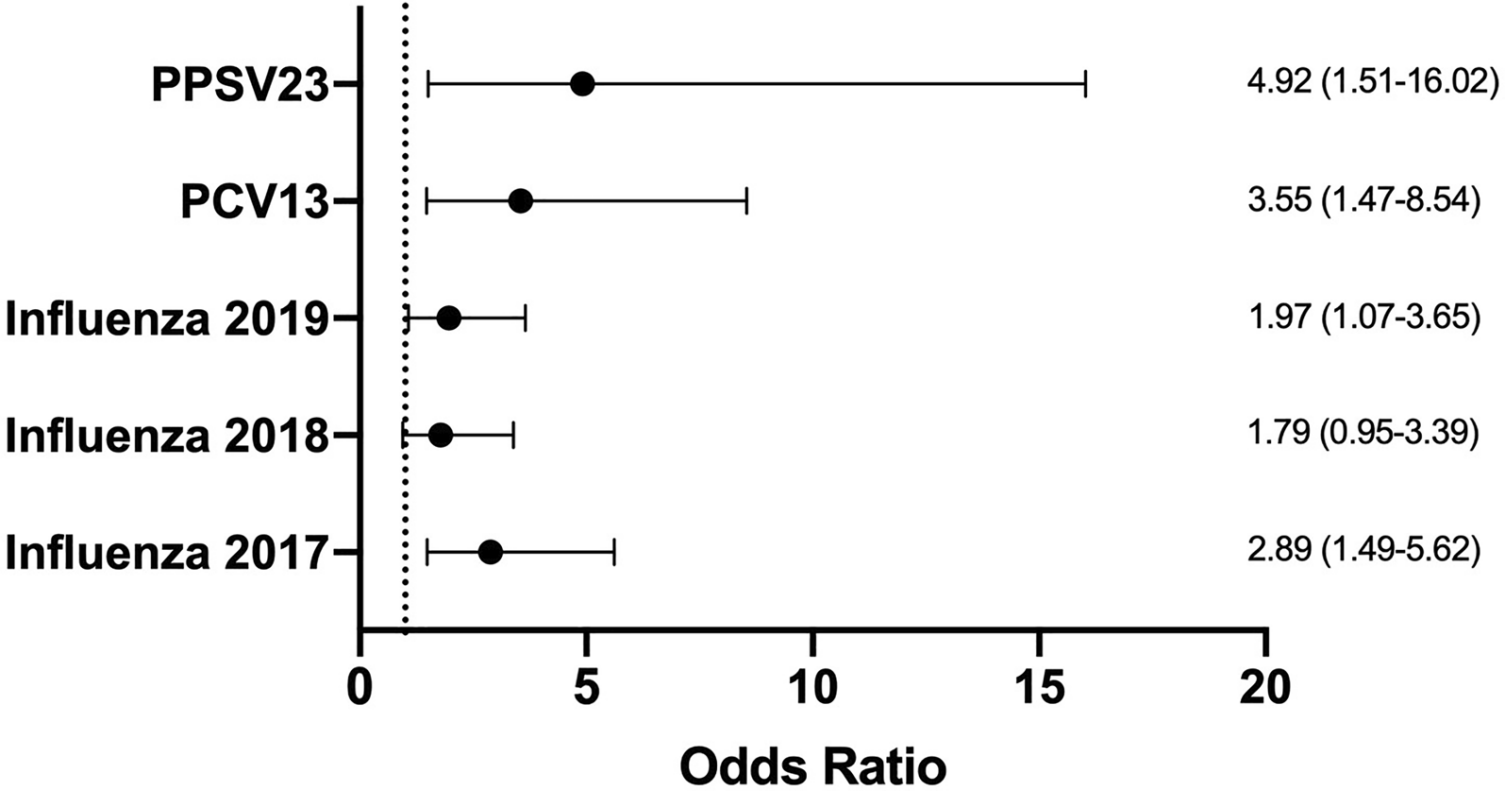
Odds ratio of pulmonary subspecilaity evalution in the prior 3 years for receiving vaccinations

In multivariable models adjusting for age, gender and smoking status, pulmonary subspecialty evaluation was independently associated with increased likelihood of vaccination adherence for each of the three vaccines (PCV13 OR = 3.55, [95% CI 1.47, 8.54]; PPSV23 OR = 4.92, [1.51, 16.02]; influenza 2017 OR = 2.89, [1.49, 5.62]; influenza 2018 OR = 1.79, [0.95, 3.39]; influenza 2019 OR = 1.97, [1.07, 3.65]) (Fig. 1).

## Discussion

In our cohort, we demonstrated persistent gaps between guideline-recommended vaccination in COPD patients and clinical care delivered. While stable COPD is most frequently managed by PC providers [14, 15], pulmonary subspecialty referral was associated with increased odds of pneumococcal and influenza vaccinations in our study. Due to disappointingly low vaccination rate for pneumococcal and influenza, these results illustrate the importance of developing strategies to improve adherence to COPD guidelines for non-pharmacological care in both the PC and pulmonary subspecialty settings.

While pneumococcal and influenza vaccinations have been shown to be effective in reducing COPD exacerbation, hospitalization and mortality of COPD patients [16, 17] vaccination rates, especially for influenza, was sub-optimal. Among patients referred to a pulmonary provider, COPD hospitalization in the year before the study period occurred for 20% in our sample. Hospitalization may mediate the likelihood of vaccination administration but ideally, vaccinations are occurring before COPD exacerbations and hospitalizations. Health illiteracy and lack of awareness regarding the importance and effectiveness of the vaccine is one of the barriers to vaccine uptake, and strategies to address these barriers have been shown to have a positive impact on influenza vaccination rate [18]. This is an area which is ripe for further quality improvement intervention that can address the challenges of campaigns for current vaccination uptake, and also anticipate the potential implications when future vaccines (i.e., SARS-CoV-2) become widely available.

There were several limitations to this study. First, we were unable to assess whether patients were managed by and receiving vaccinations from non-VA pulmonary providers. However, VA Health Factors immunization fields are routinely used by PC providers to enter reported non-VA vaccination status for patients. Second, our sample size limits our ability to detect additional, clinically significant associations and may not reflect practices of PC and pulmonary subspecialists in more diverse patient populations and/or under different payer models, but can be addressed in the subsequent studies. Third, it is unclear if the vaccinations were not offered or if patients declined the recommendations, although theoretically providers can enter when patients decline immunizations. Finally, we were unable to determine whether vaccinations were provided during pulmonary subspecialty or PC visits. It is possible that PC providers who refer patients to pulmonary may be more attuned to the COPD guideline care. Thus, partnered management of COPD by PC and pulmonology may translate into improved overall COPD care.

## Conclusion

While there remains ample opportunity to improve vaccination rates in patients with COPD, we showed patients who also received pulmonary subspecialty evaluations were more likely to receive guideline-concordant vaccinations. More studies are needed to better understand the modifiable contributors to poor adherence to vaccinations, especially in PC, and incorporating patient preferences, to improve the COPD management.

## Data Availability

The data analyzed in the current study are not publicly available.

## Abbreviations

COPD: Chronic obstructive pulmonary disease
CI: Confidence interval
GOLD: Global Initiative for Chronic Obstructive Lung Disease
ICD: International classification of disease
OR: Odds ratio
PC: Primary care
PCV13: Pneumococcal conjugate vaccine
PPSV23: Pneumococcal polysaccharide vaccine
