# Correction to: Does pulmonary subspecialty referral from primary care affect the adherence to vaccination recommendations in COPD patients?

**DOI:** 10.1186/s12931-021-01673-4

**Published:** 2021-03-15

**Authors:** Solmaz Ehteshami‑Afshar, Kristina Crothers, Benjamin Rodwin, Brett Bade, Cynthia Brandt, Kathleen M. Akgün

**Affiliations:** 1 Internal Medicine, Yale University School of Medicine, New Haven, CT USA; 2 Veterans Affairs Puget Sound and University of Washington, Seattle, WA USA; 3 Veterans Affairs Connecticut Healthcare System, West Haven, CT USA; 4 Veterans Affairs Connecticut Pain Research, Informatics, Multi-Morbidities, and Education Center, West Haven, CT USA; 5 Emergency Medicine, Yale School of Medicine, New Haven, CT USA

## Correction to: Respir Res (2021) 22:50 https://doi.org/10.1186/s12931-021-01639-6

Following publication of the original article [1], we were notified that the last author, Kathleen M. Akgün, should have also been marked as a corresponding author.

The original article has been corrected.
